# Diameter of Human Day Five Blastocysts and Birth Sex

**DOI:** 10.7759/cureus.63075

**Published:** 2024-06-24

**Authors:** Ensar Hajder, Cornelius Doehmen, Jan-Steffen Kruessel, Marco Albus, Ezz al Din Alazzeh

**Affiliations:** 1 Reproductive Medicine, University Clinic Dusseldorf, Dusseldorf, DEU; 2 Reproductive Medicine, Kinderwunschzentrum Niederrhein, Moenchengladbach, DEU; 3 Reproductive Medicine, Besins Healthcare, Berlin, DEU; 4 Reproductive Medicine, Embryology, Kinderwunschzentrum Niederrhein, Moenchengladbach, DEU

**Keywords:** pronuclei, det, set, embryo transfer, embryo, blastocyst, art, icsi, ivf, assisted reproductive therapy

## Abstract

Background

This study aimed to evaluate the offspring sex ratio, born through fresh and cryo-thawed single blastocyst (BL) transfers regarding a single morphological, static parameter, namely, BL diameter.

Methodology

This retrospective, observational study was conducted at an assisted reproductive technology (ART) center, Kinderwunschzentrum Niederrhein Germany. We conducted a statistical analysis of all births resulting from fresh and thawed in vitro fertilization (IVF) and intracytoplasmic sperm injection (ICSI) cycles after a single embryo transfer (SET). The main outcome measure was the offspring sex ratio after SET of a day five BL in relation to the BL diameter measurement.

Results

There were more female than male babies born in our study. We observed a tendency for BL to have a higher diameter, resulting in female offspring, which was not statistically relevant. We also compared the BL diameter in the fresh embryo transfer (ET) group with that of the cryo-thawed ET group, showing a tendency toward a larger diameter in the fresh ET group. In the ICSI cycles, there was a higher tendency for a larger BL diameter when compared to IVF cycles. In the fresh ET cycles, BL leading to the male sex at birth had a tendency toward a larger diameter than the female BL. In the cryo-thaw ET cycles, BL leading to the female sex had a tendency toward a larger diameter than the male BL.

Conclusions

Our results showed a tendency in the sex of offspring toward the female sex and no significant difference in the BL diameter of BL leading to birth after ART and consecutive transfer of day five BL.

## Introduction

Single embryo transfer (SET) is an effective strategy to reduce the risk of multiple pregnancies in assisted reproductive therapy (ART) [1]. Selecting the best possible embryo for embryo transfers (ETs) is one of the keys to success in ART cycles. Prolonged blastocyst (BL) culture helps the embryologist and clinician in the selection of the best embryos. In some studies, aneuploidy has been found in over 50% of BLs [2]. In many early embryos, aneuploidy is a cause of arrest. The prolonged embryo culture increases the probability of obtaining euploid embryos [3].

BL transfer results in better implantation, pregnancy, and birth rates compared to cleavage-stage ET [4-7]. Morphokinetic assessment of embryos during their development remains the gold standard in selecting the best possible embryo for success after ET. Gardner and Schoolcraft’s system is the most common and widely used scoring system for classifying embryos [4]. It is based on the assessment of the following three parameters: (1) blastocoele expansion, (2) inner cell mass, and (3) trophectoderm. To our knowledge, it is still unclear which assessment method is the best for embryo selection, yet many studies have been conducted to evaluate the impact of different BL parameters on ART outcomes [8-19]. Given this, it is important to continue research on embryo selection to further improve the quality and safety of ART.

Many studies have examined the potential confounders of morphokinetic variables such as smoking, obesity, gonadotropin dose, and stimulation protocol, as well as laboratory conditions [20-26]. However, not many studies have focused on human embryo development and birth sex. From animal studies, we know that the most rapidly growing embryos are more often male than female [27,28]. As the most developed human BLs are used for ET, one could hypothesize that the sex ratio also shifts toward the male sex. Some studies have shown faster development of male human BL while others found no significant sex differences [29-33]. Bronet et al. showed a correlation between time-lapse parameters and embryo sex in a preimplantation genetic testing (PGT) group of patients [30]. Borgstrøm et al. showed a correlation between high trophectoderm and BL development scores in favor of the male sex [33]. There are still many double embryo transfers (DETs) of embryos that have different morphokinetic parameters. Therefore, it is not always possible to predict the impact of morphokinetic parameters on implantation and birth rates. Furthermore, not always BL but earlier stages are transferred. This makes the evaluation of BL assessment criteria before an ET much more difficult. Hence, in our study, only data from SETs in in vitro fertilization (IVF) and intracytoplasmic sperm injection (ICSI) cycles were evaluated.

To our knowledge, this is one of the first studies to evaluate a single morphological static parameter in BL assessment concerning embryo sex at birth without the use of a time-lapse system. We aim to provide new insights into the selection of the correct assessment criteria for BL transfer to those who do not have an expensive time-lapse imaging system in their clinics.

## Materials and methods

In this retrospective, observational study, 100 couples treated at the reproductive clinic Kinderwunschzentrum Niederrhein in Moenchengladbach, Germany who gave birth from May 13, 2018, to September 01, 2022, were included. The focus was on the correlation between SET and the birth of one child. The mean maternal age at birth was 33.27 years (SD = 3.65). Overall, 80 of the 100 births were from ET from fresh cycles and 20 from cryo-thawed cycles. A total of 19 IVF and 81 ICSI cycles were performed, with three testicular sperm extraction (TESE)-ICSI cycles, one polar body biopsy, and one heterologous ICSI with donor sperm. All 100 female patients underwent controlled ovarian stimulation with gonadotropin-releasing hormone agonist (intranasal 4 × 1 doses Metrelef daily, Ferring) or gonadotropin-releasing hormone antagonist (subcutaneous Orgalutran once daily, Merck).

Controlled ovarian stimulation using Gonal F (Merck), Pergoveris (Merck), or Menogon (Ferring) subcutaneously was performed. The dosage of the stimulants was administered according to the individual weight, age, and ovarian reserve, the latter was estimated through sonography (antral follicle count) and anti-Mullerian hormone.

Follicular development was monitored with transvaginal ultrasound. The ovulation was triggered with Ovitrelle 0.25 mg (Merck) after three or more follicles reached 17 mm in size, and the oocyte pick-up was done 36 hours after the ovulation induction in sedation. An experienced gynecologist performed the oocyte pick-up with transvaginal ultrasound guidance.

Briefly, only for the ICSI, the cumulus-oocyte complexes were isolated from follicular fluid and then rinsed and cultured in 0.5 mL equilibrated transfer medium Global Total (Cooper Surgical, USA) and incubated at 37.0°C in 6% CO_2_, 6% O_2_, and a pH of 7.30 in a G210 K-System incubator (Cooper Surgical, USA) until ICSI insemination was performed approximately 38-40 hour post-Ovitrelle administration. Fertilization was identified by the presence of two pronuclei approximately 16-20 hours after microinjection.

For the IVF, first, the sperm was placed in the Global fertilization medium (Cooper Surgical, USA) and incubated at 37.0°C in 6% CO_2_, 6% O_2_, and a pH of 7.30. After oocyte retrieval, oocytes were placed in the medium. Ejaculated sperm was washed beforehand in a commercially available discontinuous single-layer (80%) density gradient (SpermGrad, Vitromed, Germany). On the day of oocyte pick-up, the total motile count was routinely determined after sperm processing by gradient density centrifugation. Where needed, testicular sperm was retrieved and frozen. Then, the sperm was thawed on the day of ICSI treatment. Oocytes were fertilized according to routine IVF and ICSI (with ejaculated sperm or TESE) procedures.

For IVF and ICSI, after fertilization, the fertilized oocytes were incubated in G210 incubator K-System (Cooper Surgical, USA). Global Total medium (Cooper Surgical, USA) was used for IVF until day five. The same medium was used for ICSI throughout the whole process until day five.

**Figure 1:**
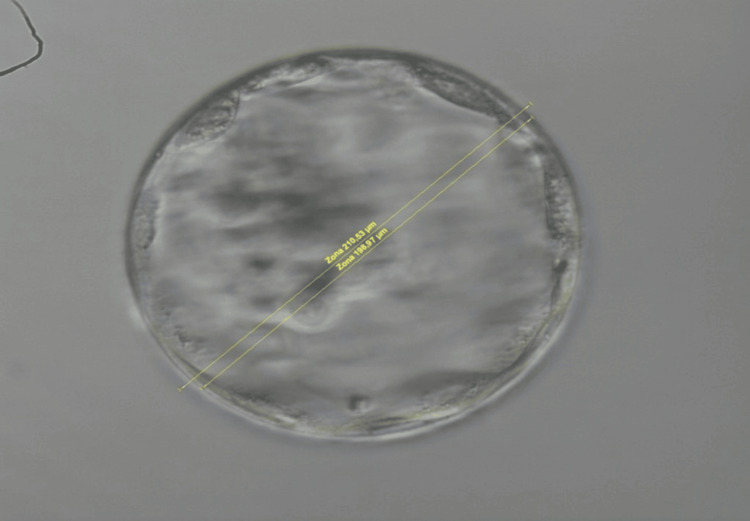
Blastocyst measurement (with and without zona pellucida) after in vitro fertilization for cryo-thaw embryo transfer.

**Figure 2:**
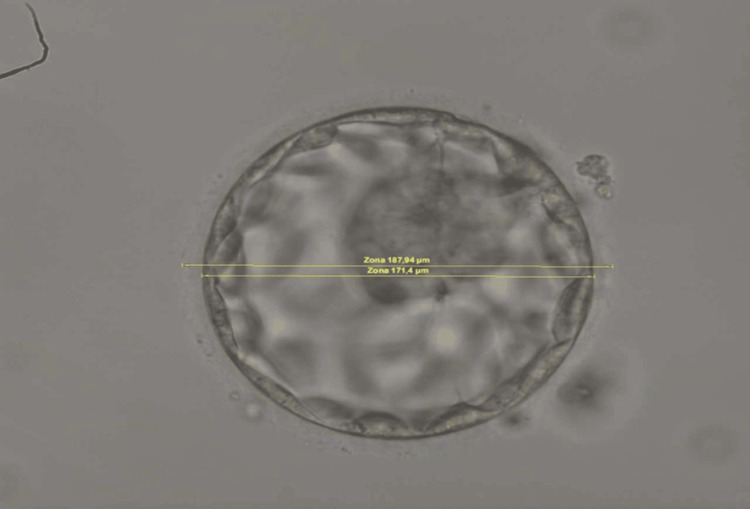
Blastocyst measurement (with and without zona pellucida) after intracytoplasmic sperm injection for fresh embryo transfer.

An experienced embryologist took a single one-plane measurement in the middle of each BL, with and without the surrounding zona pellucida (ZP) (Figures 1, 2). The latter results were used for statistical analysis. The reason behind this step was that a significant ZP width variation has been described, which can lead to false results [34,35]. The results were expressed in micrometers. The measurement was conducted with Octas computer software (Vitrolife, Sweden).

The measurements were conducted on day five 120 hours after the insemination for the fresh ET cycles. For the cryo-thaw ET cycles, the measurements were conducted 120 hours after insemination after PN and BLE thaw. Before the ET, 7 BLII were assessed 144 hours after insemination. No BLI were thawed. Of the seven BLII, three expanded and four stayed at BLII stadium.

In accordance with the study by Sciorio et al., the maximum BL diameter was used as a parameter (in this case the sole parameter) in assessing the BL and as a predictor of clinical pregnancy rates (CPRs) [36]. Only one embryo was subjected to PGT due to the female patient indication, others were not. The embryo was euploid. No other PGT cycles were indicated or performed.

The quality of BL grading was assured as a certified, experienced embryologist performed all the measurements. Secondarily, for the evaluation of all embryos in our study, we used a modified Gardner and Schoolcraft scoring system: the early BL was titled BLI, the developed BL BLII, and the expanded BL BLE. No hatching or hatched BL were considered in our study.

For the cryo-thaw cycles, embryos were frozen in an individual culture in 80 μL of the Vit Kit-Freeze medium (FUJIFILM Irvine Scientific, USA) and thawed before the ET on ET-4 (PN), ET-1 (BLII), and ET + 0 (BLE). The medium used for the thawed embryos was Vit Kit-Thaw (FUJIFILM Irvine Scientific, USA). For the fresh ET, the transfer medium Global Total (Cooper Surgical, USA) was used. The culture conditions and the medium volume used were the same for fresh and cryo-thaw cycles.

For the SET after, the prolonged BL culture Cook catheters (Cook, USA) were used. Biochemical pregnancy was investigated 14 days after transfer (ET + 14) by a blood beta-human chorionic gonadotropin test in our center, and ongoing pregnancy was confirmed by a fetal heartbeat during an ultrasound at approximately six weeks of gestation (ET + 31). The patients or the treating gynecologist gave us inputs regarding the birth and sex of the newborn approximately four to eight weeks after the birth via standardized questionnaires.

The statistical analysis was performed using SPSS version 27.0 (IBM Corp., Armonk, NY, USA). For categorical variables, Pearson’s chi-squared test was used. Continuous variables were tested for normal distribution using the Shapiro-Wilk test. The independent samples t-test was used for continuous variables with normal distribution. The Mann-Whitney U test was used for continuous variables without normal distribution. All statistical analyses were two-sided, and a p-value ≤0.05 was considered statistically significant.

## Results

This study aimed to measure the diameter of day five embryos as a morphological variable related to the sex at birth. In our study, the BL size with and without ZP had a tendency toward a larger diameter in the male population in the fresh ET group. In contrast, the BL with and without ZP in the cryo-thaw ET group had a tendency toward a higher diameter in the female group. The blastocyst in the ICSI group had a tendency toward a higher diameter when compared to the IVF group. In the fresh ET group, the BL had a tendency toward a larger diameter when compared with the cryo-thaw ET group. The findings of this study did not indicate that embryo sex at birth correlated with the BL diameter of day five BL. The correlation between maternal age and sex at birth did not show statistical significance (Table 1).

**Table 1:** Blastocyst size and sex distribution. ^c^ = unpaired t-test. BL = blastocyst; ET = embryo transfer; IVF = in vitro fertilization; ICSI = intracytoplasmic sperm injection; PN = pronuclei

BL size in µm, mean (SD)	Male	Female	P-value
Total ET (n = 100)	159.64 (24.71)	161.42 (28.85)	0.744^c^
Fresh ET (n = 80)	161.86 (23.93)	160.58 (28.39)	0.829^c^
Cryo ET (n = 20)	149.37 (27.35)	164.43 (31.57)	0.286^c^
BL size in µm, mean (SD)	IVF	ICSI	P-value
Total ET (n = 100)	156.52 (31.56)	161.58 (25.88)	0.464^c ^
BL size in µm, mean (SD)	Fresh ET	Cryo ET	P-value
Total ET (n = 100)	161.17 (26.27)	158.41 (30.16)	0.683^c^
Male (n = 45)	161.86 (23.93)	149.37 (27.35)	0.198^c ^
Female (n = 55)	160.58 (28.39)	164.43 (31.57)	0.687^c ^
BL size in µm, mean (SD)	PN cryo ET	BL cryo ET	P-value
Cryo ET (n = 20)	145.41 (23.53)	174.29 (30.86)	0.029^c^

Table 2 demonstrates the distribution of BL grades in the male and female cohorts in the total, fresh, and cryo-thaw ET groups. There were no statistically significant differences (Table 2).

**Table 2:** Blastocyst grades. ^b^ = Chi-square test. BLI = blastocyst I; BLII = blastocyst II; BLE = expanded blastocyst

Blastocyst grades			
Total (n = 100)	Male	Female	P-value
BLI	6 (42.86%)	8 (57.14%)	0.931^b^
BLII	18 (47.37%)	20 (52.63%)	
BLE	21 (43.75%)	27 (56.25%)	
Fresh (n = 80)			
BLI	4 (40.00%)	6 (60.00%)	0.912^b^
BLII	14 (46.67%)	16 (53.33%)	
BLE	19 (47.50%)	21 (52.50%)	
Cryo (n = 20)			
BLI	2 (50.00%)	2 (50.00%)	0.535^b^
BLII	4 (50.00%)	4 (50.00%)	
BLE	2 (25.00%)	6 (75.00%)	

In this study with 100 ART cycles, 45 babies were male and 55 were female. Fresh ET was performed in 80 cases and cryo-thaw ET in 20 cases. A total of 19 IVF and 81 ICSI cycles were conducted. Overall, eight babies from IVF were male (42.10%) and 11 (57.90%) were female. In ICSI cycles, 37 babies were male (45.70%) and 44 (54.30%) were female. In fresh ET, 37 (46.25%) babies were male and 43 (53.75%) were female. In the cryo-thaw, eight (40.00%) were male and 12 (60.00%) were female (Table 3).

**Table 3:** General information and sex distribution. ^a^ = Mann-Whitney U test; ^b^ = chi-square test. IVF = in vitro fertilization; ICSI = intracytoplasmic sperm injection; ET = embryo transfer

Embryo sex	Male	Female	P-value
Sample size (n)	45	55	
Maternal age in years (mean, SD)	32.89 (3.72)	33.58 (3.60)	0.348^a^
Insemination and transfer type			
IVF	8 (42.10%)	11 (57.90%)	0.778^b^
ICSI	37 (45.68%)	44 (54.32%)	
Fresh ET	37 (46.25%)	43 (53.75%)	0.613^b^
Cryo ET	8 (40.00%)	12 (60.00%)	

The mean age of the mothers in the study was 33.27 years (SD = 3.65). In the male group of born babies, the mean age was 32.89 years (SD = 3.72), and in the female group, it was 33.58 years (SD = 3.60), with no significant difference between the two groups. The mean maternal age at the time of birth of the baby after a BLE (mature and expanded blastocyst) transfer was 32.35 years (SD = 3.09), after a BLII (mature, non-expanded blastocyst) transfer was 34.05 years (SD = 4.01), and after a BLI (immature, early blastocyst) transfer was 34.29 years (SD = 3.93). Higher maternal age was associated with lower BL quality and size. In further evaluation of maternal age at birth, two groups were identified, i.e., one over 33 years of age (55 patients) and one under 33 years of age (45 patients). In the group of patients 33 years of age and older, 23 (41.82%) male and 32 (58.18%) female babies were born. In the group of patients under 33 years of age, 22 (48.89%) male and 23 (51.11%) female babies were born. In summary, there was no statistically significant correlation between maternal age at birth and newborn sex.

In this study, regardless of the embryo quality (BLI, BLII, or BLE), more female babies were born in the fresh ET cycles. There were no sex differences at birth in the cryo-thawed ET cycles after BLI and BLII transfer, and only after BLE transfer, we observed a tendency toward the female sex. The BL after BL thawing had a larger diameter than the BL after pronuclei (PN) thawing and prolonged culture (p < 0.05).

BL had a tendency toward a larger diameter in the fresh ET group compared to the cryo-thaw ET group. In the ICSI cycles, the BL had a tendency toward a larger diameter than in IVF cycles. In the fresh ET cycles, BL leading to the male sex at birth had a tendency toward a larger diameter than the female BL. In the cryo-thaw cycles, BL leading to the female sex had a tendency toward a larger diameter than the male BL. The BL had a tendency towards a larger diameter in the total female population when compared to the total male population. These results were not significant.

## Discussion

The findings of this study did not indicate that embryo sex at birth is correlated with the BL diameter of day five BL. There was no significant correlation between maternal age and sex at birth in this study.

Some studies have shown a faster development of male human BL, while others did not show any significant sex differences [29-33]. Dumoulin et al. found a sex-specific growth difference favoring the male sex in ICSI but not in IVF embryos [37]. In contrast, Bodri et al. reported that several morphokinetic parameters were associated with the female sex at the BLE stage [38]. In the study by Hentemann et al., a subgroup analysis revealed that more females were born after ICSI and fresh FET [32].

Fraire-Zamora et al. reported a statistical difference in the time to BL stage: male BL showed an increased average time to reach the start of blastulation and time to full BL stage compared to female BL [39]. found In a large meta-analysis of 1,376,454 ART cycles, Supramaniam et al. reported a predominance toward the male sex in IVF treatments compared with ICSI treatments, as well as with BL-stage ET compared with ET of cleavage-stage embryos [40].

Bu et al. conducted a multi-center retrospective study in China including 121,247 born babies. The results showed that ICSI could decrease the percentage of male offspring. BL transfer might, on the other hand, increase the percentage of male offspring [41]. Luna et al. observed a higher male sex ratio when BL transfers were performed [42]. Borgstrøm et al. showed similar results [33]. In the meta-analysis by Chang et al., a higher, statistically significant male-to-female ratio was observed after BL transfer compared with cleavage-stage ET [43]. On the other hand, other studies did not report the same results [44,45].

Perlman et al. noted that in patients in fresh ET cycles, BL-stage ET was associated with higher male sex live-birth rates compared with cleavage-stage ET [46]. Lou et al. demonstrated that a single, high-quality fresh ET at the BL stage correlates with a higher probability of resulting in male than in female offspring [47]. In the study by Mao et al., the main result was that single BL cryo-thaw ET resulted in the birth of more male babies. Transplanting good-quality BL significantly increases the proportion of born male babies [48]. Csokmay et al. showed that BL transfer did not affect the sex ratio in live births of BL with normal preimplantation screening [49].

Carrasco et al. showed that more male embryos developed to the BL stage in a retrospective study; however, more aneuploidies were observed in male BL in a PGT program [50]. In the study by Alfarawati et al., 72% of BLs attaining the highest morphologic scores were male [31]. Most studies used static criteria in evaluating embryo selection. Serdarogullari et al. focused on evaluating the effect of time-lapse criteria on the potential sex bias, and no bias was found in the study [51].

Regarding the maternal age and sex at birth, in our study, we stratified the maternal age into two categories, i.e., maternal age under 33 years and maternal age over 33 years. When comparing these two groups, there was no statistical difference in terms of sex at birth. Deignan et al. reported that more male babies were born to mothers under 34 years of age and more female babies to mothers over 37 years of age [52]. In the study by Bodri et al., the mean maternal age was 36.9 ± 3.8 years (range = 28-46 years). The study showed that the maternal age was higher in the group of women who delivered female babies [38]. Mao et al. also reported similar findings [48].

Some studies focused on the evaluation of the blastocoel expansion [8], some on the trophectoderm development [9], and others on the inner cell mass [10] for the BL evaluation and selection. The debate about which morphological parameters are appropriate in BL selection remains unclear. In accordance with the study by Sciorio et al., we measured the maximum BL diameter as a parameter in assessing the BL and as a predictor of CPRs [36].

The limitations of this study are a relatively small, unicentric cohort and the retrospective study design. The novelty of our study is that, to our knowledge, this is the first retrospective study from a single center to analyze the offspring sex ratio after a single transfer of day five BL in terms of a single morphological parameter: the measurement of BL diameter in one plane without using time-lapse. The results might prove useful for embryologists and clinicians who do not have the expensive time-lapse equipment in their clinics but are still looking for new ways to assess BL morphology.

## Conclusions

To our knowledge, this is the first retrospective, single-center study analyzing the offspring sex ratio after a single transfer of day five BL in relation to a single morphologic parameter, i.e., the BL diameter measurement. In our study, a tendency toward a higher birth rate of female babies, regardless of the embryo quality (BLI, BLII, or BLE), was observed in the fresh ET cycles. In the cryo-thaw ET cycles, after BLI and BLII transfer, there were no sex differences at birth, only after BLE transfer, we saw a tendency toward the female sex. In the fresh ET cycles, BL leading to the male sex at birth had a tendency toward a larger diameter than the female BL. In the cryo-thaw cycles, BL leading to the female sex had a tendency toward a higher diameter than the male BL. BL had a tendency toward a higher diameter in the total female population when compared to the total male population. In the cryo-thaw cycles, the BL after BL thawing had a significantly larger diameter than the BL after PN thawing and subsequent prolonged culture. No statistical significance was shown in the results when correlating maternal age with sex at birth. In conclusion, further multicenter studies with a larger patient cohort and the same criteria are needed to further investigate this issue.
